# Applied artificial intelligence for global child health: Addressing biases and barriers

**DOI:** 10.1371/journal.pdig.0000583

**Published:** 2024-08-22

**Authors:** Vijaytha Muralidharan, Joel Schamroth, Alaa Youssef, Leo A. Celi, Roxana Daneshjou

**Affiliations:** 1 Department of Dermatology, Stanford University, Stanford, California, United States of America; 2 Faculty of Population Health Sciences, University College London, London, United Kingdom; 3 Stanford Center for Artificial Intelligence in Medicine and Imaging, Department of Radiology, Stanford University, Stanford, California, United States of America; 4 Laboratory for Computational Physiology, Massachusetts Institute of Technology, Cambridge, Massachusetts, United States of America; 5 Division of Pulmonary, Critical Care and Sleep Medicine, Beth Israel Deaconess Medical Center, Boston, Massachusetts, United States of America; 6 Department of Biostatistics, Harvard T.H. Chan School of Public Health, Boston, Massachusetts, United States of America; 7 Department of Biomedical Data Science, Stanford University, Stanford, California, United States of America; University of Ulm, GERMANY

## Abstract

Given the potential benefits of artificial intelligence and machine learning (AI/ML) within healthcare, it is critical to consider how these technologies can be deployed in pediatric research and practice. Currently, healthcare AI/ML has not yet adapted to the specific technical considerations related to pediatric data nor adequately addressed the specific vulnerabilities of children and young people (CYP) in relation to AI. While the greatest burden of disease in CYP is firmly concentrated in lower and middle-income countries (LMICs), existing applied pediatric AI/ML efforts are concentrated in a small number of high-income countries (HICs). In LMICs, use-cases remain primarily in the proof-of-concept stage. This narrative review identifies a number of intersecting challenges that pose barriers to effective AI/ML for CYP globally and explores the shifts needed to make progress across multiple domains. Child-specific technical considerations throughout the AI/ML lifecycle have been largely overlooked thus far, yet these can be critical to model effectiveness. Governance concerns are paramount, with suitable national and international frameworks and guidance required to enable the safe and responsible deployment of advanced technologies impacting the care of CYP and using their data. An ambitious vision for child health demands that the potential benefits of AI/Ml are realized universally through greater international collaboration, capacity building, strong oversight, and ultimately diffusing the AI/ML locus of power to empower researchers and clinicians globally. In order that AI/ML systems that do not exacerbate inequalities in pediatric care, teams researching and developing these technologies in LMICs must ensure that AI/ML research is inclusive of the needs and concerns of CYP and their caregivers. A broad, interdisciplinary, and human-centered approach to AI/ML is essential for developing tools for healthcare workers delivering care, such that the creation and deployment of ML is grounded in local systems, cultures, and clinical practice. Decisions to invest in developing and testing pediatric AI/ML in resource-constrained settings must always be part of a broader evaluation of the overall needs of a healthcare system, considering the critical building blocks underpinning effective, sustainable, and cost-efficient healthcare delivery for CYP.

## Introduction: Interconnected problem drivers

At the heart of pediatric global health is the goal of delivering sustained, holistic, and equitable progress in child health and development outcomes [1]. In recognition of the growing role played by artificial intelligence and machine learning (AI/ML) within healthcare, it is important to consider how these technologies can support the advancement of global health objectives for children and young people (CYP) [2,3].

Despite significant interest and emerging studies in pediatric healthcare AI/ML, we remain in the foothills of exploring the potential and implications of these digital health technologies [4]. Existing research papers exploring machine learning in pediatrics span early disease detection and diagnostics, prognostic scoring systems based, risk prediction models, disease classification systems, and a range of clinical decision support tools [2,4–8]. Data types for training models include electronic medical record (EMR) data, imaging data such as chest radiographs or neuroimaging (i.e., electroencephalogram data), and even digital biomarkers for diagnosing cognitive impairment based on speech. A range of machine learning approaches have been used, including neural networks, deep learning, regression algorithms, Bayesian algorithms, decision tree algorithms, clustering algorithms, natural language processing, and ensemble methods [2]. However, this field is still at an early stage, with the literature consisting primarily of proof-of-concept studies. There are comparatively few studies evaluating algorithms actually deployed within pediatric practice and current clinical care, although this is expected to change in the coming years as AI/ML systems are further integrated clinical practice [2,4,8].

Several review articles focused on global use cases for pediatric AI/ML highlight how research is, thus far, overwhelmingly undertaken in high-income settings, typically in academic centers [2,7]. A 2021 systematic review found that 82% of the available literature on child and adolescent healthcare ML was from high-income countries (HICs), while only 3% of studies came from lower middle-income countries [2]. This finding reveals a potential misalignment, because 90% of the global child and adolescent population are based in lower and middle-income countries (LMICs) [9]. Other review articles have focused on healthcare AI/ML applications within LMICs for all age groups, and these identified few papers focused on pediatric use cases [10–12]. Notably, a 2022 systematic scoping review of AI/ML systems deployed in LMICs included only a single pediatric use-case, while a review article focused on AI/ML for pediatric tuberculosis noted that of the 21 articles included, only 2 papers related to algorithms validated on data sets that included patients under 15 years old [5,10]. In summary, there is a major shortfall in published clinical studies on the use of pediatric AI/ML tools in LMICs.

If the potential benefits of technological progress are to be realized, advances in healthcare AI/ML and their application must be equitably distributed and representative of the needs of pediatric populations worldwide, in order both to support universal progress in child health and avoid worsening of existing disparities [12].

This narrative review explores a number of pediatric-specific challenges relating to healthcare AI/ML with a particular focus on LMICs, discussing how these might be overcome so that AI/ML can positively impact global child health. These issues include governance and ethics, as well as key CYP-specific technical considerations and in the opinion of the authors, this topic demands urgent attention and prioritization for clinicians, software developers, and policymakers [13].

Governance, ethics, and privacy concerns are central to the use of CYP data in healthcare AI/ML. In the United States, the FDA has approved medical devices that include children among the target population [14]. However, there remains a critical need for laws, policies, guidelines, and incentive structures that account for age-specific considerations and complexities in the pediatric population, catering to variations in age, development, disease type, and geographical location so that CYP are both protected and included as healthcare AI/ML progresses forward.

Delivering equitable healthcare AI/ML for children globally requires recognition of intersecting challenges that include (i) the additional practical and ethical complexities inherent in pediatric research; (ii) the chronic underrepresentation of LMICs in healthcare research globally; and (iii) challenges inherent in health technology research in lower-resource settings [15,16]. Furthermore, general well-characterized pitfalls of healthcare AI/ML research and deployment (data quality, drifts and shifts, algorithmic bias, and poor generalizability) also require attention in this context [8,17–19].

Beyond AI/ML, children remain underrepresented in clinical research efforts globally compared to adults in both high- and low-income settings, due to a range of structural factors [16]. The problem of representation is greatest in those from traditionally marginalized groups, lower socioeconomic backgrounds, ethnic minorities, patients with rare diseases, and LMICs [15]. These aforementioned challenges can be considered together as interconnected problem drivers that present barriers for progress in child health globally, and we call for interdisciplinary healthcare AI/ML teams developing tools for CYP must recognize and confront this complexity throughout the research and development lifecycle [13].

### Technical and methodological challenges: Using pediatric data

The SPIRIT-AI and CONSORT-AI guidelines established for clinical trial design and reporting present a framework for regulation and policymaking in healthcare AI [20,21]. While these guidelines are comprehensive for study design and reporting across specialties, they do not account for specific age-related considerations and ethical complexities that AI/ML research presents in children.

Recently, pediatric AI best practice (ACCEPT-AI) was drafted by bioethicists, researchers, and clinicians, although consensus is still needed to represent diverse viewpoints [13].

ACCEPT-AI outlines 6 key areas that must be prioritized to promote equity and minimize bias and harm, including age-specific considerations, consent and assent, communication, equity, and technological considerations. ACCEPT-AI has been designed to complement existing formalized guidelines such as SPIRIT-AI and CONSORT-AI, but places emphasis on the specific challenges of “age-related algorithmic bias,” which refers to systematic errors that result from the discrepancy between adult and pediatric data in AI data sets [13]. The failure to segregate data by age and account for heterogeneity in physical and cognitive development of children in health data sets can lead to inappropriate generalizations of pediatric data to an adult population and vice versa. Recent examples include evaluation of an AI algorithm trained to interpret echocardiograms that found differences in model performance between adult and pediatric data, with superior performance in adults compared to children [22]. The authors concluded that child and adult data sets should be considered separately in training and testing to optimize model performance. Similarly, a dermatology study highlighted the importance of training and testing algorithms on pediatric data for the detection of melanoma and improving the overall generalizability of dermatology AI models [23].

Even in high-income settings, there is simply less data for pediatric populations than adults, resulting in more limited EHR data sets for training models [8,24]. These smaller data sets are themselves highly heterogeneous due to the variations that occur within normal ranges and variation in disease presentation and treatment approaches for children of different age groups, posing a number of technical challenges to data quality and variety [7,24]. With limited training samples comes greater statistical noise, potentially undermining predictive capability.

Irregularity in temporality within EHR data risk undermining model performance, particularly in pediatrics as this issue is compounded by the fact that children undergo major developmental progress in their early years, impacting both their normal physiology, behavior, and disease manifestations [8]. While strategies exist for handling different types of missing data, temporally irregular data has been found to pose challenges for predictive capabilities of deep learning algorithms trained on longitudinal EHRs [25].

Additional issues relate to how algorithms are successfully deployed and evaluated in pediatric populations and how adverse events and algorithmic harm specific to children and young people (captured either in clinical studies or routine deployment of an algorithm) must be addressed [8,13,24]. Issues of bias and generalizability are paramount and are discussed in the following section.

Finally, challenges in the broader pediatric research landscape risk negatively impacting AI/ML efforts. The ethical and legal complexities of pediatric research, logistical and technical difficulties of studying medical interventions in children, and comparatively less funding of pediatric clinical research may all affect the quantity and quality of training data sets [16]. In the opinion of the authors, recognizing this reality is essential when developing strategies to undertake and deliver equitable, effective healthcare AI/ML for CYP throughout the AI/ML lifecycle [26].

### Barriers and biases: Representative pediatric data sets for ML research globally

In high-stakes AI/ML domains such as healthcare, large training data sets representative of target populations are essential. Issues with data quality or validity can lead to major downstream risks, such as the need for significant technical updates and model iterations, or worse, project abandonment and harm to beneficiaries [10,27].

When considering pediatric data sets in both high-, middle-, and low-resource settings, major differences and variation in healthcare systems, clinical practice, sociocultural norms, human resources, and EHR use between and within HICs and LMICs pose crucial challenges to algorithmic performance and the generalizability of ML systems [3,15]. Within the US, training data originates predominantly from only a handful of states, typically from large academic centers, posing technical challenges to model generalizability and raising concerns around inclusion and equity [28]. This same phenomenon is replicated globally, with data sets originating predominantly from a small number of HICs, while both adult and pediatric populations in LMICs are underrepresented [2].

This lack of LMIC population representation in training data sets is multifactorial. The comparatively limited use of electronic health records and less mature digital infrastructure in some MIC or LIC settings will inevitably play a role [2,10]. Naturally, “LMICs” are a highly heterogeneous group—technically “middle” income nations such as India and Brazil have large EHR data sets at national, regional, and local levels [10]. By comparison, in lower middle-income or low-income countries, sufficiently large, complete, and regularly updated data sets for training models may be not available, very limited, or may be viewed as inadequate for the purposes of AI/ML training, introducing the potential of geographical bias [29,30]. Even where data collection is routinely undertaken, challenges in the processes for digitization, data management, and underlying IT infrastructure can inhibit potentially rich sources of clinical data [10].

Where EHRs are in use, patient records will reflect the realities of clinical practice on the ground. For example, in a large systematic review of global pediatric AI/ML, the pediatric intensive care unit (PICU) was frequently the setting for clinical research with AI/ML, as it presents a rich repository of clinical data for training models, typically focused on clinical decision support and early detection [2,7]. However, PICUs are overwhelmingly based in HICs and tertiary academic centers, undermining the relevance of such tools in settings where few PICUs exist.

The role of power dynamics and socioeconomic inequalities also shapes data sets. Through determining what data are included and excluded and the chosen measurements for capturing the ground truth, data sets (and the model outputs that follow) are unavoidably a product of the socioeconomic, cultural, political norms, and agendas of that context, reflecting existing power structures [27].

The systemic underrepresentation of specific populations, groups, or regions within training data has been termed “health data poverty” [31]. When the chronic underrepresentation of LMIC and pediatric populations in training data sets intersects with the aforementioned challenges of using pediatric data in AI/ML models, the issue of health data poverty as it relates to CYP may be further compounded and risks perpetuating global child health inequities [32].

### Ethics, safety, and governance

Concerns regarding the oversight of AI/ML for pediatrics include questions over healthcare data privacy and governance in addition to age-specific vulnerabilities inherent to children and young people whose minds and bodies are still developing. Issues such as CYP-specific agency and consent in making healthcare decisions and the role of the parent or carer in the overall care and health of children are central. However, a recent systematic review of global standards for AI ethics failed to identify specific ethical frameworks focused on CYP, highlighting an urgent gap [33,34]. As summarized by UNICEF, “Children interact with or are impacted by AI systems that are not designed for them, and current policies do not address this” [35].

Ethicists have proposed that AI/ML algorithms must be grounded in clinical needs of CYP and attuned to the pediatric population to ensure the core pillars of medical ethics, the principles of autonomy, beneficence, non-maleficence, and justice are upheld [36,37].

Beneficence may be determined by ensuring that algorithms are developed to positively impact child health priorities with clearly defined outcomes linked to pediatric health and wellbeing. The principle of non-maleficence can be incorporated by ensuring that potential harms (linked to privacy, data security, technical issues) that may arise through technology development are accounted for, monitored, and mitigated as appropriate [13,35,38].

The core ethical principles of autonomy and justice will be supported by the appropriate, equitable, and diverse participation of young people globally in AI/ML initiatives, grounded in the logic of human-centered AI (discussed in the following sections) to help address concerns over fairness and justice [38–44]. Scholars have also proposed a fifth ethical tenant for AI, “explicability,” which aims to complement the other principles, encompassing both understanding the decisions made (the “black box” problem) but also making explicit who is accountable for model outputs, both of which can be considered paramount in healthcare for CYP and in LMICs [10,36].

A review of global guidelines for AI Ethics found that published recommendations were highly concentrated in Europe and North America while largely absent in the rest of the world [33]. With this status quo, it is inevitable that choices around ethics largely reflect western norms, yet it cannot be assumed that western norms and values around algorithmic bias, fairness, inclusion, and privacy are automatically portable across different contexts in LMICs [33].

Since “fairness” (a concept linked to “justice”) has no single definition that would apply across pediatric AI/ML for children globally, teams developing algorithms will need to establish clear aims and objectives so that models can be tested and adjusted as required, affirming the crucial need for experts in child health and welfare within AI/ML teams [35,39]. This will inevitably involve trade-offs when competing measures of fairness and justice for children must be considered and weighted against each other.

Pediatric AI/ML grounded in ethical principles must be supported by robust, coordinated, and fit-for-purpose governance structures. Global governance of healthcare AI/ML medical devices is in its infancy and remains fragmented, with limited coordination of laws that differ across nations, particularly in the context of data protection and removal, which are crucial considerations for child protection [45].

Policymaking organizations are increasingly recognizing the importance of establishing a hierarchy of accountability and international standards for the potential misuse of technology that may compromise child protection [13,35]. These standards must be robust and also adaptive, remaining fit-for-purpose as the role of AI/ML in CYP healthcare evolves [38]. However, currently there is no clear consensus and further efforts are required.

At the local and regional level, Institutional Review Board (IRB) processes and consent laws vary across nations, as do approaches in CYP consent [46]. Greater harmonization and standardization of policies across borders and moving towards globally generalizable regulation, safety and governance will be an essential step for the safe and equitable inclusion of children and pediatric data for AI/ML in HICs and LMICs alike.

Developing pediatric-specific international standards is paramount. Emerging coordinated efforts such as the “Pediatric Moonshot” aim to unify data across pediatric hospitals internationally to develop safe and generalizable algorithms through an internationally federated lab and represent an important step towards global coordination of AI/ML for the pediatric population [47].

While western nations and organizations such as the OECD and the EU have so far led in setting standards and governance frameworks for healthcare AI, the “Global South” is increasingly developing its own standards and frameworks. Organizations such as the Africa-Asia AI Policymaker Network was established in 2022 to bring together policy and regulatory leaders to develop frameworks and strategies for responsible AI/ML globally that include diverse regional viewpoints [48]. Examples include the African Academy of Sciences and the African Union Development Agency recommendations for coordinated data governance for participant-centered research involving human participants, while the Science for Africa AI and Data Science initiative is a holistic attempt to convene scientific, government, civil society, and industry representatives for improved AI/ML governance in Africa, with a focus on global health [49,50]. The Africa-Canada Artificial Intelligence and Data Innovation Consortium is one example of an emerging health data partnerships between HIC and LMICs [15].

### Co-design: Equity, inclusion, and education

Inclusion is central to addressing several key challenges facing global pediatric healthcare AI/ML. In the simplest sense, inclusivity requires broadening training data to include CYP from a broad range of backgrounds to ensure that historically marginalized groups of CYP, such as those from ethnic minorities or those with developmental delay, are not excluded from technological efforts that have the potential to benefit them [32].

However, inclusion refers not only to which populations in the training data for models, but also who is involved in developing and validating these tools [38,43]. UNICEF guidance on AI and children emphasizes the importance of partnering with young people to co-design innovations [35]. In the US and UK, researchers have engaged with CYP on the topic of healthcare AI, demonstrating through education programs that CYP generally express a strong interest in and desire to be involved in the design, policy-making, and implementation for healthcare AI [40–42]. Such papers highlight how CYP recognize the importance of this topic and want “a seat at the table” including through being consulted on model inputs and outputs, at a high level so that they have a voice. Similarly, a WHO framework on “youth-centered” digital health interventions called for CYP advisory boards to input into healthcare projects, with meaningful involvement at every stage of the research and development lifecycle [38,51]. Conversely, the failure to incorporate CYP voices into the development of novel healthcare technologies carries significant risks to their viability, feasibility, and sustainable deployment within healthcare systems [43,44]. Digital and AI literacy initiatives for children can empower CYP to support these aims, ranging from education in technical understanding of coding AI/ML algorithms, the explainability of the algorithm’s output, and human–computer interaction [35,39]. International consensus on educational objectives around AI and ML will help steer towards inclusive AI/ML design for health [35,38]. However, dialogues must consider and address the digital literacy divide and the gap in the availability of expertise and technological resources between regions and countries [10].

Participatory co-design of health AI tools, “with children and for children” is already happening, albeit in HICs (for example, a large CYP mental health AI project in Helsinki) [52]. Inclusive co-design requires engaging with CYP, their families, and communities as partners at all stages project inception through to development, deployment, and embedding of healthcare AI/ML solutions [39,44,53]. Ensuring this approach can be adopted globally, including in low-resource settings, requires a deliberate and proactive approach. Partnering with Community Led Organizations (CLOs) may help increase population engagement through trusted networks in specific communities, enabling inclusion of “hard-to-reach” groups and overcoming data poverty [54]. This is particularly important for communities who may typically be excluded from healthcare through poverty or discrimination, such as indigenous populations [55,56]. Community Advisory Boards (CABs) are a mechanism for supporting the research process in low-income settings, adapting research tools to best fit local contexts, being agile in recognizing and meeting community needs [57]. Another approach termed Community-Based Participatory Research (CBPR) ensures communities partner with researchers to engage in any decisions regarding study designs and conduct and ensure this meets their needs—an explicit empowerment of these community voices in the research process [54]. Partnering with children and their communities in Healthcare AI research in LMICs entails complexities and upfront investments, but this approach is fundamental if healthcare AI/ML tools for children are to be designed in a way that can have a positive, equitable impact globally [57].

### Implementation science and human-centered AI

Human-centered AI (HAI) proposes that the design of intelligent AI/ML systems must recognize the algorithm’s roles and interactions as part of a broader system consisting of human stakeholders [58]. This approach to both research and implementation entails acknowledging and accounting for the nuanced, often culturally specific, expectations and needs of humans in return helping humans understand these AI systems, explaining the AI/ML outputs in ways that nonexpert end users can interpret and use in their shared common goals [58].

HAI is therefore an interdisciplinary and collaborative endeavor that takes on additional complexities when considering (i) healthcare AI/ML in low-income settings; and (ii) healthcare AI/ML for pediatric populations.

When technical experts developing AI/ML models are siloed in HICs, working separately from child health domain experts on the ground in LMICs, this can undermine the utility and value of the model once deployed [53,59]. This is particularly important given that cutting edge healthcare AI/ML systems Healthcare AI research in are currently typically developed in HIC, or by teams led by those in HICs, outside of the context in which the model may one day be deployed. The principles of HAI require that healthcare teams obtain meaningful knowledge of the complexities and nuances of local health systems and clinical workflows to understand and build around how end users (typically healthcare professionals) interact with and adopt these digital tools [60]. In low-resource settings, this means placing greater value on local knowledge and inputs [12]. A paper focusing on AI/ML practitioners in India and East Africa identified that flawed assumptions about end users relating to healthcare literacy, language barriers, or IT infrastructure and can lead to incorrect interpretation of model outputs that undermine and complicate existing workflows and cause stress to both healthcare workers and patients [10,27]. Grounding AI/ML systems for pediatric care within the realities of existing clinical workflows, structures, and processes can help overcome the difficulty of the “last mile” implementation with end users, which has so often limited the adoption and therefore success of AI/ML tools, particularly in LMICs [10,61]. An illustrative case is that of a deep learning system developed in HICs but deployed in an LMIC clinic for retinal disease, where issues with clinic infrastructure, internet connectivity, staff and patient behavior all drastically undermined the potential benefit of the algorithm [60].

In relation to pediatrics, HAI necessitates broad, interdisciplinary expertise. Child psychologists, pediatricians, nurses, child-rights experts, and ethicists may be integrated into AI/ML research teams alongside user experience professionals (UX), human–computer interaction (HCI), human-factors engineers, and implementation scientists [62]. As outlined previously, children and their families can play a key role as co-creators in this wider team depending on the type of tool being developed [38,43]. Technical teams developing models need a baseline level of literacy relating to critical issues of welfare and rights [39]. For AI/ML developers and research teams, understanding how to ensure the inclusion and welfare of children in the context that the algorithm is deployed must be considered core knowledge.

Implementation phases of healthcare algorithms for CYP must continuously evaluate hard clinical endpoints and weigh them against the risk of harm, including unintended consequences [2,7,8]. Accountability, responsibility, hierarchies, and power balance are currently under-explored in this field, particularly with algorithms that impact CYP. However, established mechanisms such as provision of data sheets and model cards, mechanisms for community engagement, and open multi-stakeholder dialogue at each step of the AI lifecycle can provide vital safety components of the AI pipeline to ensure those who are disproportionately burdened by disease benefit the most [63,64].

### Building capacity for AI/ML within health systems

While applied pediatric AI/ML within LMICs remains in its infancy, researchers and clinicians are increasingly aiming to address this knowledge gap as LMICs further invest in IT infrastructure, continue to adopt EHRs and technological capabilities improve globally [2,10]. It is encouraging that a growing body of literature has demonstrated proof of concept in key pediatric global health areas such as childhood malaria. These include a number of papers validating predictive and diagnostic algorithms on blood films for malaria in West African nations [65–67]. Diagnostic algorithms for detecting tuberculosis from chest radiographs and pneumonia classification from ultrasound images holds promise for application to pediatric subjects, while areas of potential promise also include diagnosis of childhood anemia [5,68–73]. AI/ML enabled smartphone apps have highlighted the potential role of AI/ML in supporting the prevention of childhood malnutrition in India and general health promotion [74].

Further encouraging developments include the growth of data sharing and research networks in LMICs dedicated to inpatient pediatric clinical research, for example, a cohort of hospitals in Mozambique and other sub-Saharan African countries [75]. Such networks will be essential for data sharing and creation of high-quality, standardized data sets for training ML models.

While these proof-of-concept AI/ML use cases demonstrate real potential, testing and deployment are essential. Without implementation and evaluation of the real-world performance of these algorithms in low-resource settings (and their impact on key outcomes of interest), the proposed benefits of AI/ML for global pediatrics will remain unrealized. Translation from proof-of-concept mandates deployment within the chosen clinical context and ongoing monitoring and evaluation for safety and efficacy [10].

To realize this ambition, there is consensus that it is crucial to develop the capabilities and infrastructure for pediatric AI/ML globally [12,15,38]. Pragmatic proposals include increased investment in the technical training of developers in LMICs, adequately compensating and improving training for community health workers who collect data and ensuring that clinical staff in lower-resource settings has the time, incentives, and ability to participate in the co-design and evaluation of AI/ML systems for CYP [27]. Increased training of researchers and developers in LMICs is key so that the next generation of healthcare AI teams are drawn from diverse, globally representative AI/ML workforce with a variety of lived experiences and backgrounds in high- and low-resource settings, attuned to the needs and context of the communities in which these algorithms are deployed [10,15].

Ultimately, AI/ML investment and funding decisions must first be part of a broader evaluation of the overall needs of a healthcare system and the relevant resource constraints, considering the critical building blocks underpinning effective, sustainable, and cost-efficient healthcare delivery [76]. Healthcare AI/ML investment must make sense as part of a broader consideration of, and commitment to, system development and investments aligned to meeting the greatest needs of the world’s CYP [77].

Both human and technical factors here are essential. There is an irony that with increasing focus on AI/ML for low-resource settings, the reality remains that some low-income countries (and also low-income regions within middle- or high-income countries) may be struggling with fundamentals required to support these technologies, such as reliable internet coverage, basic cybersecurity, and even dealing with power outages [77]. Worse still, the enthusiasm for how AI/ML may improve health systems and outcomes in LMICs may end up distracting policymakers, researchers, and clinical leaders from investments in more pressing needs and priorities, such as improving critical healthcare infrastructure and improving services for children [10,76]. Training staff and building capacity in AI/ML knowledge and skills in LMICs will be important, yet this must not come at the expense of the training and upskilling of staff in delivering the fundamental clinical services for CYP.

## Conclusions

Currently, there are very few AI/ML tools applied to pediatric care outside of high-income countries, limiting the potential of AI/ML to address the global burden of pediatric disease. This narrative review has discussed how various barriers can be overcome in order for AI/ML to have a positive impact on child health in high- and low-resource settings. Firstly, there must be greater emphasis on the unique CYP-specific challenges at different stages of the AI/ML life cycle, with broader recognition of these issues and adoption of CYP-specific standards for data. In lower-resource settings, fundamental challenges to both healthcare and AI/ML research and deployment are well established. CYP can be considered a subgroup in these populations where extra attention and concerted effort is required.

Governance, regulatory, and policy frameworks can enable a positive, equitable vision of AI/ML for CYP; however, these require open dialogue and consensus to deliver guidance that can be effective and useful beyond high-income settings and facilitate safe and effective collaborations globally. Safeguarding CYP and mitigating harms and risks is paramount. Addressing valid concerns around inclusion and equity, AI/ML for CYP must be human-centered and (where appropriate) co-designed with CYP and their caregivers, who must be considered critical stakeholders in this process. Integrating child health expertise into interdisciplinary healthcare AI teams and ensuring these technologies meet the needs of the children and communities they are intended to benefit must become standard practice in both high- and low-income settings. Data sharing, research collaborations, and development outside of HICs is essential. Finally, equitable investment, capacity building, and education globally is essential to enable global impact of healthcare AI/ML for children and young people. While the focus of this paper has been AI/ML, broader investment in electronic healthcare records and general health system capacity building for CYP is an essential step that will underpin any potential benefits of emerging AI/ML tools. Developing safe, effective, and equitable AI/ML for CYP globally can act as a shared objective around which a wide range of stakeholders can unify, advocate, and accelerate research, deployment, and evaluation.

### Limitations

We acknowledge the limitations of the paper, primarily that this is a perspective-based narrative review. While we appreciate that a systematic or scoping review would provide a higher level of evidence, a sparsity of studies at this intersection limits this possibility, which would be considered for future work. Plans are in process to gain expert consensus in this area.
